# Bacterial thiol oxidoreductases — from basic research to new antibacterial strategies

**DOI:** 10.1007/s00253-017-8291-8

**Published:** 2017-04-13

**Authors:** Katarzyna M. Bocian-Ostrzycka, Magdalena J. Grzeszczuk, Anna M. Banaś, Elżbieta Katarzyna Jagusztyn-Krynicka

**Affiliations:** Department of Bacterial Genetics, Institute of Microbiology, Faculty of Biology, University of Warsaw, Miecznikowa 1, 02-096 Warsaw, Poland

**Keywords:** Dsb (disulfide bond), Virulence, Secretion system, Antibacterial drugs

## Abstract

The recent, rapid increase in bacterial antimicrobial resistance has become a major public health concern. One approach to generate new classes of antibacterials is targeting virulence rather than the viability of bacteria. Proteins of the Dsb system, which play a key role in the virulence of many pathogenic microorganisms, represent potential new drug targets. The first part of the article presents current knowledge of how the Dsb system impacts function of various protein secretion systems that influence the virulence of many pathogenic bacteria. Next, the review describes methods used to study the structure, biochemistry, and microbiology of the Dsb proteins and shows how these experiments broaden our knowledge about their function. The lessons gained from basic research have led to a specific search for inhibitors blocking the Dsb networks.

## Introduction

Cysteine residues play a crucial role in post-translational modification that guarantees correct protein folding. The oxidation reaction between two cysteine thiol groups, leads to formation of a disulfide bond. While disulfide bonds can form spontaneously in the presence of atmosphere, the reaction is slow. Instead, in vivo disulfide bond formation is catalyzed by a range of proteins, the thiol oxidoreductases of the Dsb (disulfide bond) system. The first Dsb protein discovered was *Escherichia coli* DsbA (Bardwell et al. 1991). Since then, the Dsb protein network of *E. coli* (EcDsb) has been well-characterized through a combination of microbiological, biochemical, biophysical, and proteomic approaches. Several excellent review papers presenting the details of the process have recently been published (Berkmen 2012; Cho and Collet 2013; Denoncin and Collet 2013; Kadokura and Beckwith 2010). To briefly summarize, in *E. coli*, the activity of the periplasmic and soluble monomeric DsbA generates disulfides in a consecutive manner on polypeptide chains that are translocated into the periplasm, whereas dimeric periplasmic EcDsbC is responsible for shuffling improperly formed disulfides. Correct functioning of the EcDsbA and EcDsbC is ensured by two inner membrane proteins (EcDsbB and EcDsbD, respectively). EcDsbB converts reduced EcDsbA back to the oxidized form by transporting electrons to either ubiquinone or menaquinone. The integral membrane protein EcDsbD keeps EcDsbC in the reduced form by catalyzing the transfer of electrons from the cytoplasm to the periplasm.

Outside of *E. coli,* other bacteria have extremely diverse Dsb systems, both in terms of the numbers of proteins playing a role in the introduction of disulfide bonds, and in their structures and interactions. We still do not fully understand why some bacterial species need incredibly complicated sets of Dsb proteins, while others thrive with exceedingly simple systems. Significant differences in Dsb systems have been observed between species of the same genus as well as between strains of the same species (Bocian-Ostrzycka et al. 2015a; Grimshaw et al. 2008; Lin et al. 2009). A growing number of sequenced bacterial genomes makes it difficult to enumerate all the Dsb systems described so far. Some were depicted in two review papers published some years ago (Heras et al. 2009; Lasica and Jagusztyn-Krynicka 2007). The number of Dsbs that are oxidants vary among bacterial species. Some possess several DsbA proteins with different substrate specificities that interact with one or more DsbBs, while others have only a single homolog of DsbA and DsbB (Arts et al. 2013; Heras et al. 2010; Sinha et al. 2004). Dsb system diversity also involves the redox partners of periplasmic thiol oxidoreductases. A majority of Dsb oxidases are converted into the oxidized form by proteins homologous to EcDsbB. However, in some bacteria, this function is taken over by DsbI or VKOR proteins. DsbI operates in only a small number of bacteria; it is homologous to the DsbB family and consists of two domains. Its N-terminal domain, consisting of five transmembrane helices, resembles classical DsbB, whereas its periplasmically-located C-terminal adopts a β-propeller structure (Lasica et al. 2010). VKOR is a bacterial homolog of mammalian vitamin K epoxide reductase, which is a functional equivalent of EcDsbB (Dutton et al. 2008; Wang et al. 2011). Thiol oxidoreducases playing a role in rearrangements of improper disulfides are kept in reduced forms by at least three structurally similar, but not identical, inner membrane proteins: DsbD, ScsB or CcdA (Cho and Collet 2013; Cho et al. 2012; Katzen et al. 2002; Stirnimann et al. 2006a). It was noted several years ago that *E. coli* cells lacking DsbA and DsbB are still able to generate disulfides. Recently, this function was assigned to a periplasmic protein containing one cysteine residue, PspE, which in cooperation with DsbC, is able to at least partially replace DsbA/DsbB (Chng et al. 2012).

Though most of the thiol oxidoreductases that act as oxidants are monomeric; the list of dimeric thiol oxidoreductases has lengthened (Bocian-Ostrzycka et al. 2015b; Daniels et al. 2010; Kpadeh et al. 2013, 2015). Most of these dimeric thiol oxidoreductases, described so far, act as isomerases and form homodimers. They interact with two redox partners of different structure (DsbD or ScsB) (Cho et al. 2012; Jiao et al. 2013; McCarthy et al. 2000). One of the most complex Dsb systems operates in *Legionella pneumophila* cells. It consists of two DsbAs (monomeric and dimeric), two DsbBs, and two DsbDs. Interestingly, this microorganism does not possess DsbC and uses dimeric LpDsbA2 not only to form disulfide bonds but also to correct improperly introduced disulfide bonds (Kpadeh et al. 2013, 2015). On the other hand, *Helicobacter pylori* lacks both classical DsbA/DsbB and DsbC/DsbD homologs. Instead, it uses two untypical Dsb proteins to generate disulfides: HP0231 and HP0377. HP0231 is a dimeric oxidase and HP0377 is an aberrant CcmG (*cytochrome c maturation*). In contrast to other CcmGs described so far, HP0377 is bifunctional, acting not only in the reduction of apocytochrome c but, additionally, in disulfide isomerization. HP0377 exists as a mixture of monomeric and dimeric forms (Bocian-Ostrzycka et al. 2015b, 2016; Lester et al. 2015; Roszczenko et al. 2012, 2015).

As mentioned above in Gram-negative bacteria, the process of protein oxidative folding takes place in the periplasm, whereas in Gram-positive bacteria, lacking this compartment, it occurs in the space between the cytoplasmic membrane and the cell wall (Chagnot et al. 2013). Thiol oxidoreductases involved in disulfide generation of some Gram-positive bacteria have been also characterized in regard to their mechanism of action, biochemical features, and structures (Daniels et al. 2010; Heras et al. 2008; Ishihara et al. 1995; Kouwen et al. 2007).

Finally, it should be stressed that even though overwhelming numbers of bacterial species generate disulfide bonds in the periplasm, there are some thermophilic microorganisms belonging to both archeal and bacterial domains that contain many proteins with disulfides in reducing cytosolic compartments. These bacteria, which live under extremely harsh conditions, use this post-translational modification for protein stabilization (Jorda and Yeates 2011; Ladenstein and Ren 2006).

The first part of the paper presents current knowledge of how Dsb systems affect virulence of many pathogenic microorganisms. Next, the review paper presents the strategies used for structural and biochemical characterization of the Dsb proteins that provide insights into details of the mechanisms of their actions. The rapid increase of bacterial antimicrobial resistance in recent years has become a major public health concern in many countries. Many substrates of Dsb system in pathogenic bacteria are extracytoplasmic proteins that are involved in virulence. Thus, the detailed knowledge about Dsb network functioning has enabled initial research to make the Dsb network the target for a new class of antivirulence drugs.

## Influence of the Dsb system on virulence — impact on secretion systems and defense against stress conditions

Pathogenic bacteria produce different virulence factors. A majority of them are extracytoplasmic proteins with varied localization, which ensure adhesion, pathogen survival, and replication inside host cells or modulate the functioning of the host immune system. Thus, bacterial pathogenicity is strictly dependent on correct functioning of the secretion pathways. A classification of secretion systems based mainly on evolutionary and functional relatedness is not completely clear. Gram-negative bacterial secretion systems are classified into six or nine groups designated by Roman or Arabic numerals (Chagnot et al. 2013; Green and Mecsas 2016). In general, proteins are transported by a one- or two-stage process. The first uses complex transporter machinery to deliver effector molecules directly from the bacterial cytoplasm into the cytosol of a wide range of eukaryotic cells. In two-stage transport, proteins are first delivered to the periplasm through the bacterial inner membrane, and next they are transported across the outer membrane using various mechanisms.

**Fig. 1:**
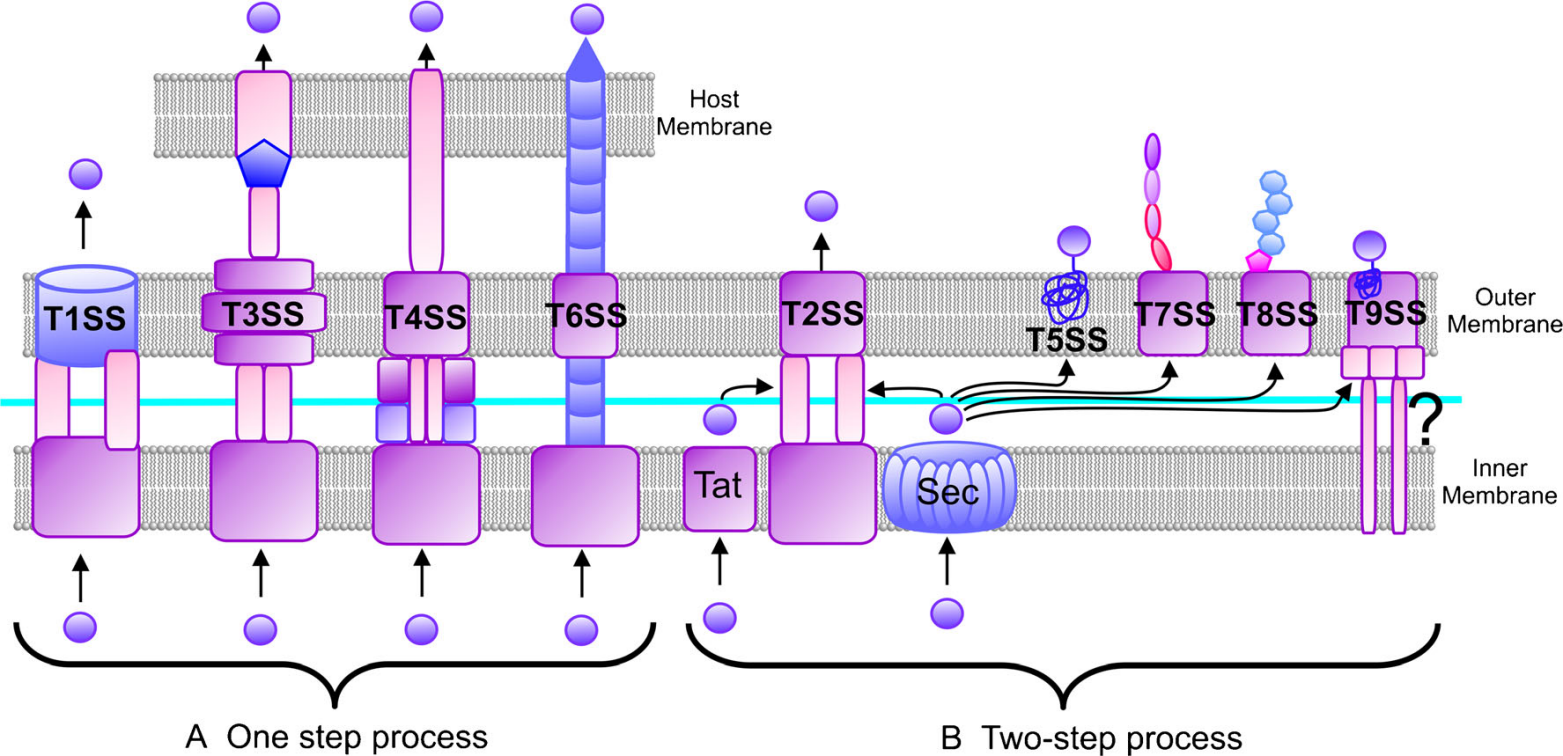
Secretion systems of Gram-negative bacteria. **a. ** One-step systems (*T1SS*, *T3SS*, *T4SS*, *T6SS*). In these systems effector molecules, mainly proteins, are delivered directly to the cytosol of a target cell. They are transported through a secretion channel that spans three membranes: the inner and outer bacterial cell membranes and the membrane of a eukaryotic cell. The architecture of the secretion apparatus of each type of system is unique. **b. ** Two-step systems (*T2SS*—pathway previously named GSP (general secretion pathway), involved also in the formation of Type 4 pili, *T5SS*—pathway used by autotransporters which do not need any auxiliary proteins to traverse the outer membrane; *T7SS*—chaperone-usher pathway involved in pili formation; *T8SS*—extracellular nucleation-precipitation pathway involved in curli formation; and *T9SS*—Por (porphirin) secretion system. In these systems proteins cross the inner membrane with the help of either the Sec (secretion) or Tat (twin arginine transportation) pathway and next they are transported across the outer membrane using various mechanisms (for details see Chagnot et al. 2013 and Green and Mecsas 2016)

This chapter will focus on reviewing the role of various secretion pathways in virulence. Emphasis will be given to the impact of the Dsb network on the correct functioning of the process. We present a few examples that document how Dsb protein activity affects both the structures of effector molecules and/or the structures of a wide variety of proteins comprising the macromolecular complex of transport machineries—types of secretion systems occuring among Gram-negative bacteria are shown in Fig. 1.

In the case of T3SS (type three secretion system), T4SS, and also T6SS, effector molecules, mainly proteins, are transported through a secretion system channel that spans three membranes: the inner and outer bacterial cell membranes and the membrane of a eukaryotic cell (Fig. 1a). Molecules that are delivered into host cells are able to convert the physiology of those cells in a way that is advantageous for the pathogen. The transporters of these three systems are complex organelles, sometimes made up of more than 20 proteins (Backert and Meyer 2006; Galan and Wolf-Watz 2006; Moraes et al. 2008). In T3SS, the targets of the Dsb systems are not the effector molecules, which do not transit through the periplasm, but rather, the targets are protein building blocks of the transporter. As a majority of DsbAs show rather broad specificity and react with many substrates, their activity impacts the structures of various proteins involved in the generation of transport machineries. For example, DsbAs are required for the correct folding of *Yersenia pestis* YscC and *Shigella flexneri* Spa32. Both proteins are components of the type 3 secretion apparatus (Jackson and Plano 1999; Watarai et al. 1995). *Salmonella enterica* possesses two T3SS associated with virulence that are encoded by *Salmonella* pathogenicity islands 1 and 2 (SPI1 and SPI2). DsbA *Salmonella* mutant strains show decreased secretion of effectors via the SPI1 T3SS or the SPI2 T3SS. So far, there is no confirmed in vivo target protein for DsbA in the SPI1 T3SS apparatus (Lin et al. 2008). In contrast, SpiA, an outer membrane component of *Salmonella*’s SPI2 T3SS, requires disulfide bond formation to gain correct function (Miki et al. 2004). Additionally, in many pathogenic bacteria the Dsb system plays a role in the regulation of expression of genes involved in T3SS functioning. For example, a *Pseudomonas aeruginosa dsbA* mutant strain shows reduced T3SS secretion of effector proteins (exoU and exoT). Expression of *exoT* is regulated by transcriptional activator ExsA, which is not expressed in a *dsbA* mutant (Ha et al. 2003; Lin et al. 2008).

T4SS secretion machinery is also Dsb-dependent. A *Helicobacter pylori* strain that does not produce HP0231, the main dimeric thiol oxidoreductase responsible for disulfide bond formation, is avirulent because it does not translocate CagA (cytotoxin associated gene) into gastric epithelial cells (Roszczenko et al. 2012; Zhong et al. 2016). This phenotype potentially results from the wrong conformation of CagL, which contains one disulfide bond and acts as a T4SS adhesion protein (Barden et al. 2013; Conradi et al. 2012). *Legionella pneumophila* DsbA2 (a bifunctional dimeric thiol oxidoreductase) is necessary for proper assembly and function of the T4SS that is essential for invasion and intracellular replication of this pathogen. Several proteins involved in assembly of the secretion apparatus were captured using mutated DsbA2 (P198T) (Jameson-Lee et al. 2011; Kpadeh et al. 2015).

The type 6 secretion system is a one-stage, contact-dependent delivery machinery similar to the type 3 and 4 secretion systems. T6SS has been found in more than 25% of bacterial genomes sequenced so far. In two aspects, T6SS is different from other one-stage secretion pathways. First, type 6 has a different evolutionary origin. Secondly, in contrast to others one-stage secretion systems, it ensures direct contact not only between the pathogen and host cells but sometimes between bacterial cells. Thus, it also plays a role in bacterial antagonism, the competition for a specific ecological niche (Hachani et al. 2016). To the best of our knowledge, there is only one documented example of the influence of the Dsb network on components of the type 6 secretion system. Qin et al. showed that an atypical Dsb of *Francisella tulariensis*, FipB (FtDsbA), which displays oxidase, isomerase, and chaperone activity, affects the virulence process by influencing proper assembly of the type 6 transport apparatus (Qin et al. 2016).

The ability to adhere to a wide range of surfaces is crucial for many pathogenic bacteria. Specific cell organelles, such as adhesion pili located on the cell surface, are involved in the process. Biogenesis of *E. coli* pili (named chaperone-usher pathway, T7SS) is a well-characterized process that includes transport of all necessary elements via the inner membrane to the periplasm, followed by pilus construction on the cell surface (Lillington et al. 2014). In the periplasm, the proteins necessary for pili assembly, are protected from aggregation and degradation by specific, immunoglobulin–like chaperones: PapD (type Pap pili) or FimC (type 1 pili). The proper assembly of *E. coli* pili is strictly dependent on disulfide generation in pilus subunits or in chaperones (Crespo et al. 2012; Jacob-Dubuisson et al. 1994). Two of the three *Neisseria meningitidis* DsbAs, lipoproteins that atypically are anchored to the inner membrane are involved in the biogenesis of type IV pili. Furthermore, one of the three DsbAs of *Salmonella typhimurium*, SrgF, is indispensable for the correct assembly of a plasmid-encoded pili, PefA (Bouwman et al. 2003; Tinsley et al. 2004).

T2SS (Fig. 1b), previously named GSP (general secretion pathway), is a two-stage system that actively participates in transport of broad array of substrates, mainly enzymes (Green and Mecsas 2016). One of them is *Vibrio cholerae* toxin (CT), the main virulence factor of this pathogen. Inactivation of the gene encoding TcpG (the *V. cholerae* homolog of DsbA) results in avirulence. A strain that is not able to generate disulfides is deficient in colonization because it does not produce toxin co-regulated pili (TCP) and does not secrete cholera toxin. CT belongs to the family of AB_5_ toxins, composed of one A subunit (CT-A) with enzymatic activity and five B subunits (CT-B) responsible for toxin transport across the outer membrane and for recognition of specific receptors on the surface of eukaryotic cells. Absence of TcpG results in rapid degradation of the CT-B (Peek and Taylor 1992; Yu and Kaper 1992). Additionally, TcpG has an impact on expression of the main *V. cholerae* transcription activator ToxT, through influencing the structure of two transmembrane regulators, TcpP and ToxR, that contain two cysteine residues in their periplasmic domains (Fengler et al. 2012; Morgan et al. 2015).

The type 5 secretion system, including proteins called autotransporters (AT), is unique in its architecture and mechanism of action. ATs do not need any auxiliary proteins to traverse the outer membrane as their C-terminal translocation domains form specific beta barrel channels in the outer membrane, ensuring transport of the passenger domain to the cell surface or into the environment (Wells et al. 2007). All autotransporters described so far are virulence factors with diverse functions. Most contain a pair of cysteine residues present near the C-terminus of the passenger domain that are targets of the Dsb systems. While lack of Dsb proteins does not strongly interfere with activity of the autotransporters, the Dsb proteins generate disulfide bonds during their periplasmic transit. The process increases stability of the autotransporters and renders them resistant to protease digestion (Bodelon et al. 2009; Brandon and Goldberg 2001; Letley et al. 2006).

Often, but not always, inactivation of genes that encode periplasmic, soluble thiol oxidases results in loss of mobility, and this has a significant impact on virulence. Flagella biogenesis is a complicated, strictly regulated process. In some species, the lack of disulfide bond generation affects the structure of FlgI (P-ring flagellar protein) (Bardwell et al. 1991; Turcot et al. 2001). However, even for strains with homologs of FlgI that do not possess cysteine residues, the knockout of *dsb* genes sometimes results in loss of motility, indicating that other essential proteins, besides FlgI, for biogenesis of this organelle are also Dsb dependent (Grabowska et al. 2014).

Bacteria often have to survive unfavorable environmental conditions, such as high temperature, high oxygen concentrations, or high concentrations of various metals. Pathogenic bacteria are exposed to harmful reactive oxygen species (ROS), produced not only by their own metabolic processes but also by the host immune system as the bacteria attempt to colonize. Bacteria have therefore evolved many defense mechanisms to circumvent the lethal effect of ROSs. The defense mechanisms fall into two groups: those inactivating ROS and those involved in repairing damaged proteins (Atack and Kelly 2009; Guo and Gross 2014; Wang et al. 2006). Effective defense requires reductive activity, making the Dsb proteins essential participants in the protection process. The Dsb proteins involved in electron transport from cytoplasmic thioredoxin to the periplasm (DsbD or ScsB) constitute the hub of the defense, as shown by numerous examples. Bacteria with knockout *dsb* genes are sensitive to paraquat or hydrogen peroxide (Achard et al. 2009; Lester et al. 2015). Using *Caulobacter crescentus* as a model organism, Cho et al. surprisingly showed that peroxides are scavenged in the cell envelope by coordinated action of reductive pathway proteins, operating in the oxidative periplasm environment. That coordinated action has three components: membrane ScsB transports electrons from the cytoplasm to a periplasm-located thioredoxin-like TlpA, which subsequently delivers them to peroxidoredoxin PprX. PprX reduces ROS before they can reach cytoplasm (Cho et al. 2012). In *E. coli*, the interaction between AraF (L-arabinose–binding protein) and EcDsbC demonstrates another defense mechanism against oxidative stress. EcDsbC protects single cysteine residues against oxidative stress and is involved in reducing disulfide-linked dimers generated by AraF under oxidative stress conditions (Denoncin et al. 2014). In *Neisseria gonorrheae,* PilB (multifunctional polyprotein), which also depends on DsbD transporting reducing equivalents from the cytoplasm, plays a role in repairing oxidized methionine residues (Brot et al. 2006).

Copper (Cu), a trace metal, is an essential micronutrient for eukaryotic and prokaryotic organisms. However, an excess of copper is cytotoxic under aerobic and anaerobic conditions, and diverse bacterial Cu detoxification systems contribute to pathogenesis (Djoko et al. 2015). Since copper is an oxidant and catalyzes the generation of non-native disulfides, the defense against copper stress is achieved by the action of thiol oxidoreductases with isomerase activity. (Hiniker et al. 2005). Besides the well-characterized EcDsbC, the periplasm-located, homodimeric thiol oxidoreductase ScsC of *Salmonella typhimurium*, which is encoded by the four-gene locus *scsA-D* (copper sensitivity), is also essential for copper stress protection (Shepherd et al. 2013).

## How to distinguish between thiol oxidoreductases functioning as oxidants or reductants

In vivo, the thiol oxidoreductases involved in disulfide generation are kept in oxidized forms by interaction with homologs of the EcDsbB proteins. On the contrary, Dsbs that function as isomerases are kept in reduced forms. These two forms are undistinguishable using standard SDS-PAGE because they migrate through the polyacrylamide gels at the same speed. Thus, the protein redox states in vivo are examined by modifying their free cysteine residues using AMS (4-acetamido-4′-maleimidylstilbene-2,2′-disulfonic acid) or MalPEG (polyethylene glycol (PEG)-conjugated malemide). Both reagents react with free thiol groups to produce a major mobility shift for the modified protein in SDS-PAGE gels (Denoncin et al. 2013; Kpadeh et al. 2013). This method, known as “AMS trapping”, allows determination of the redox state of proteins.

Absence of Dsbs involved in oxidative pathways often results in pleiotropic effect because they are proteins of rather low specificity. Thus, generation of isogenic mutants and examination of their phenotypes can be a useful strategy to understand their function. However, because some DsbAs possess high specificity, this strategy sometimes does not permit to identify the effect of gene inactivation by simple checking cell physiological features. It is noticeable especially for bacteria that encode more than one thiol oxidase (Arts et al. 2013; Jameson-Lee et al. 2011). In recent years, the Dsb network of the model microorganism *Escherichia coli* has been characterized in detail (Denoncin and Collet 2013; Shouldice et al. 2011). To understand how the uncharacterized thiol oxidoreductases function, it is therefore helpful to examine their role in *E. coli* by complementation analysis. For complementation, the gene of interest is cloned into an appropriate plasmid, usually under an inducible promoter, and introduced into *E. coli* mutants for *dsbA* or/and *dsbC*. Many phenotypic traits can be checked by standard procedures, including: motility, Cd^2+^ or Cu^2+^ resistance, mucoid phenotype and alkaline phosphatase activity (Cho et al. 2014; Dumoulin et al. 2005; Grabowska et al. 2014; Hiniker et al. 2005; Leverrier et al. 2011; Roszczenko et al. 2012, 2015).

The next standard step in analysis of thiol oxidoreductases involves protein purification and determination of the protein’s biochemical attributes. The insulin reduction assay is a widely used, standard method to measure oxidoreductase activity. It specifically probes disulfide reductase activity. Insulin contains two intramolecular disulfide bonds that connect the A and B chains. The reduction of these disulfide bonds results in precipitation of the B chain which can be monitored by following absorbance at 650 nm. This strategy reflects protein reducing activity. Thus the oxidant EcDsbA has only about 10% of the activity of EcDsbC isomerase. Some thiol oxidases of narrow specificity display an even lower activity, as compared to the standard EcDsbA (Arts et al. 2013; Collet et al. 2002; Grabowska et al. 2014; Heras et al. 2008; Holmgren 1979a, b).

The next characteristic feature of thiol oxidoreductases is the redox potential of their CXXC motifs. This is determined from the equilibrium constant with glutathione; the method used depends on the presence or absence of tryptophan residues, using either a fluorometric measurement or evaluating the ratio of reduced and oxidized protein forms by the AMS trapping method (Arts et al. 2013; Lafaye et al. 2009; Roszczenko et al. 2012, 2015). In general terms, the numerous thiol-disulfide oxidoreductases are classified into three types in terms of their redox potentials: low, mid or high potential. Cytoplasmic thioredoxins involved in maintaining protein cysteine residues in their reduced form are characterized by low potentials. Disulfide isomerase proteins of mid redox potential play a role in shuffling incorrect disulfides, whereas the high potential thiol oxidoreductases located outside of the cytoplasmic membrane are involved in disulfide generation. An important characteristic of thiol-disulfide oxidoreductases is the lowered p*K*a of the reactive cysteine, which determines reactivity in thiol-disulfide exchange reactions. A clear relationship exists between the p*K*a values of the active-site cysteine residues and their redox properties: the lower the p*K*a value of the N-terminal cysteine residue, the higher (less negative) the reduction (redox) potential (Edeling et al. 2004; Lewin et al. 2008).

To distinguish between oxidizing and isomerase activities, two substrates are employed in most cases: RNaseA from bovine pancreas or hirudin, a small thrombin inhibitor that is secreted by leeches to let them freely suck blood. In their native forms, RNaseA contains four disulfides and hirudin contains three disulfides. The substrates are used in two forms: reduced or scrambled. Thiol oxidoreductases acting as oxidants display activity only with reduced forms of the substrates, whereas isomerases catalyze both disulfide bond isomerization of scrambled proteins and protein oxidation (Bocian-Ostrzycka et al. 2016; Chim et al. 2013; Hiniker et al. 2007; Kurz et al. 2008; Messens et al. 2007; Quan et al. 2007; Ren et al. 2009).

## Identifying Dsb protein substrates

Substrate identification has been crucial in understanding the role of Dsb systems in bacterial pathogenicity. A data based search for proteins with at least one cysteine pair is the initial step to identify Dsb substrates via in silico methods. Results show that cysteine residues are more frequent in cytoplasmic proteins compared to those residing outside the cytoplasm. Additionally, a majority of extracytoplasmic proteins contain an even number of cysteine residues. This trend was named “up and down”, which means, for example, that the number of proteins containing four cysteine residues outnumbers those carrying three cysteine residues. Thus, the extracytoplasmic location of proteins with even numbers of cysteine residues is an indication of their Dsb-dependence (Daniels et al. 2010; Dutton et al. 2008). Also, in silico structural modeling supplies informative data about the presence of consecutive or nonconsecutive disulfides. Recently, several algorithmic techniques have been elaborated to determine the presence of disulfides in a protein of interest (Becker et al. 2013; Lin and Tseng 2010; Marquez-Chamorro and Aguilar-Ruiz 2015; Singh 2008).

The results obtained by in silico methodology are confirmed by in vivo or in vitro methods, through global analysis of proteins present in cell-lysates or experiments conducted with purified proteins. The first analysis that identified *E. coli* and *S. enterica* Dsb targets included comparative proteomic experiments (comparison of periplasmic subproteomes derived from wt and *dsb* mutated strains (Agudo et al. 2004; Hiniker and Bardwell 2004). Originally, it was accepted that proteins containing disulfides are generally periplasmic. However, it has recently been shown that many outer-membrane proteins are also Dsb-dependent (Leverrier et al. 2011; Ruiz et al. 2010). Thus, Arts et al. in their comparative proteomics experiments with *Pseudomonas aeruginosa* wt and Pa*dsbA*1-mutated strains used proteins derived from both cellular compartments, the periplasm and the outer–membrane, which identified more than 20 previously unknown PaDsbA1 substrates (Arts et al. 2013).

The mixed complexes between EcDsbA and its substrates are short-lived and are difficult to detect. The CXXC and cis-Pro loop are highly conserved motifs characteristic for thiol oxidoreductases. It has been shown that mutations in the *EcdsbA* gene, which alter the conserved *cis*-proline, facilitate isolation of EcDsbA complexes and allows identification of its substrates. The P151T mutant of EcDsbA slows the second step of oxidative folding, which results in accumulation of EcDsbA complexes with substrates (Kadokura et al. 2004, 2005). Similarly, to trap and purify the substrates linked to a Dsb protein, the C-terminal cysteine of CXXC catalytic site may be replaced by a serine or alanine. These mutated proteins are able to react with their substrates but lack the second cysteine of the catalytic motif required to resolve the mixed disulfide complex. The proteins of cells containing a mutated version of Dsb are then analyzed by SDS-PAGE, with or without reducing agent. Usually, several additional proteins appear after reducing treatment, as compared to the non-reducing condition. They are cut out of the gel and analyzed by mass spectrometry. This method was used to identify EcDsbC substrates by Denoncin et al., EcDsbG targets by Depuydt et al., *Plasmodium falciparum* thioredoxin substrates by Sturm et al., and *Francisella tularensis* FtDsbA targets by Ren et al. and Qin et al. (Denoncin et al. 2010, 2014; Depuydt et al. 2009; Qin et al. 2016; Ren et al. 2014; Sturm et al. 2009). The mutated Dsb proteins can also be used to identify their substrates in experiments performed in vitro. In this test, purified Dsb proteins with an appropriate mutation and a His-Tag are immobilized on a Ni-NTA agarose column, followed by loading with wild type strain lysate. Dsb complexes are eluted and analyzed by SDS-PAGE under reducing and non-reducing conditions. The Dsb substrates are then identified by mass spectrometry (Ren et al. 2014).

## Dsb proteins as targets of new antibacterial drugs

The discovery of antibiotics and their introduction into common practice was one of the most important medical achievements of the previous century. However, euphoria was brief. Rapid emergence of multiple-drug resistant bacteria has increasingly occurred in many parts of the world and constitutes a serious threat to public health and patient safety. According to the European Centre for Disease Prevention and Control (ECDC) (ECDC 2014), WHO (World Health Organization) (WHO 2014), or the CDC (Centers for Disease Control and Prevention) (CDC 2013), each year infections caused by a subset of resistant bacteria are responsible for about 25,000 deaths in Europe and more than 20,000 in USA. The problem of resistance involves both Gram-positive and Gram-negative pathogens that cause infections in the hospital and in the community. Success at fighting infectious disease will depend upon development of new, effective, and safe antimicrobial compounds. More than 20 novel classes of antibiotics were introduced into the market between 1930 and 1962. Since then, only two new classes of antibiotics have been introduced (Bassetti et al. 2013; Boucher et al. 2009; Ventola 2015a, b). In recent years, scientists have implemented new strategies to grow microbes found in soil, and these approaches resulted in identification of two new antibacterial substances: teixobacin and lungdunin (Ling et al. 2015; Zipperer et al. 2016). One widely explored approach to generate new classes of antibacterial drugs focuses on targeting virulence rather than the viability of bacteria. Numerous proteins or processes have been selected as targets of therapeutic value. In general, the new inhibitors can be classified into two categories: those blocking a single virulence factor, such as adhesin or toxin, and those with a global activity to block major bacterial processes such as quorum sensing, the two-components system, secretion systems or post-translational protein modification (Brackman and Coenye 2015a, b; Duncan et al. 2012; Felise et al. 2008; Kalia 2013; Krachler and Orth 2013).

**Fig. 2:**
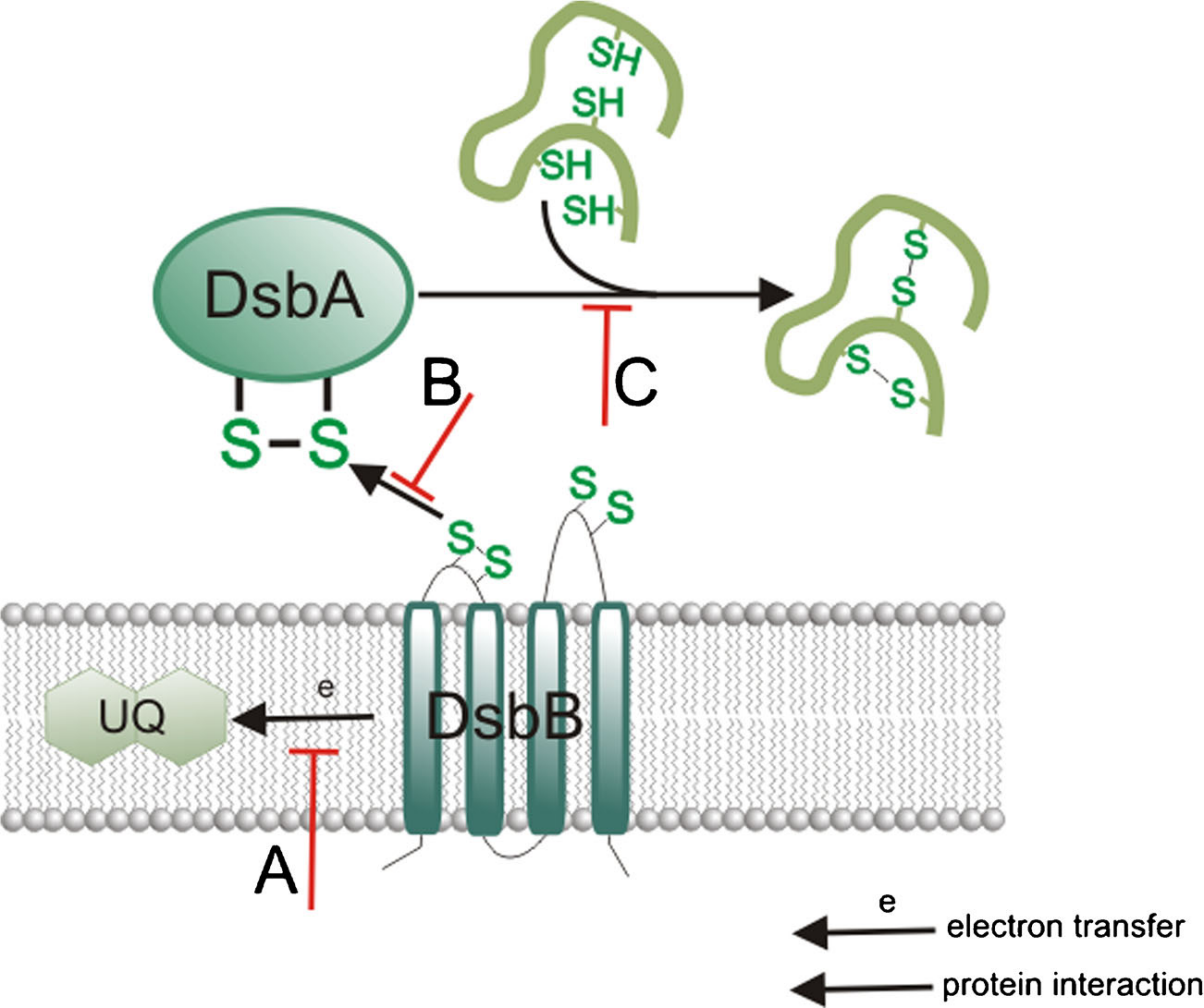
Potential sites of action of Dsb system inhibitors. **a.** Blocking interaction of EcDsbB with ubiquinone, **b.** blocking interaction of EcDsbA with its substrates, and *c* blocking EcDsbA reoxidation by EcDsbB

Proteins of the Dsb system, which play a key role in the virulence of many pathogenic microorganisms, represent possible new drug targets. Inhibition of their interactions with substrates or their redox partners could constitute a means of blocking the formation of virulence factors. The location of the Dsb proteins (in the bacterial periplasm) renders them easily accessible to potential small-molecule inhibitors. Potential sites of action of Dsb system inhibitors are shown in Fig. 2. Furthermore, use of a Dsb-protein inhibitor should not create selective pressure for developing resistance. However, it should be noted than some Dsbs have homologous proteins in eukaryotes, so inhibitors need to be vetted in detail for any detrimental effects in vertebrates.

While enormous progress has been made in understanding the mechanisms of Dsb proteins, their interdependence and the diversity of Dsb networks among bacteria, as well as the influence of Dsbs on virulence, structural biology is a key factor for initiating research on therapeutic application of Dsb inhibitors. Most interest has been directed towards solving the structures of homologs of EcDsbA proteins because they are responsible for disulfide generation. The number of crystallographic studies of DsbA homologs has increased at lightning pace, with 76 structures of DsbA proteins now deposited in the Protein Data Bank (PDB). Although all monomeric DsbAs share essential structural features (presence of the thioredoxin fold, with CXXC and cis-pro motifs), they differ substantially in charge distribution on the surface and structural attributes in the region surrounding active site. These subtle differences find reflection in dissimilarities in their biochemical properties and specificity for various substrates. Comparison of the structures and biochemical attributes of 13 DsbA homologs grouped them into two classes that subsequently, based on surface features, were divided into four subclasses (McMahon et al. 2014). Solving the structure of the EcDsbA/EcDsbB complex indicated which fragments of both proteins played a role in their mutual interaction, and this was a starting point to search for inhibitors of the process (see below) (Inaba et al. 2006; Zhou et al. 2008). Structures of homodimeric Dsbs (DsbC and DsbG), have been also solved, and this facilitates understanding of their functioning (Heras et al. 2004; McCarthy et al. 2000). Additionally, knowledge about the complicated process of transporting reducing power from the cytoplasm to the periplasm has recently deepened (Stirnimann et al. 2006a, b; Williamson et al. 2015). Although current research on inhibitors of the Dsb system mainly concentrates on disulfide generation, it is probable that blocking enzymes involved in rearranging improper disulfide bonds might also provide positive results.

The identification of small molecules working as inhibitors of virulence is a lengthy and expensive process. With the growing availability of drug libraries and automatic robotic systems, the first step to search for Dsb inhibitors involves high throughput screening (HTS). This approach rapidly tests large numbers of compounds. HTS usually requires a specific strategy to detect inhibitor activity on analyzed proteins, such as construction of fusion proteins for in vitro or in vivo tests or specific techniques to catch direct interaction of an inhibitor with purified protein/s. The complementary approach to look for Dsb inhibitors makes use of in silico analysis combined with the solved structure of a protein. They are referred as SBVS (structure-based virtual screening) and SBDD (structure-based drug design), or RTDD (rational target-based drug design). Further in silico studies aimed at structural analysis of potential drugs enables introduction of chemical modifications that may lead to more effective compounds.

So far, potential Dsb system inhibitors have been identified by both HTS and SBVS techniques. Landeta et al. searched for compounds interacting with EcDsbB by in vivo HTS. For this approach, the authors applied a specific agar, multi-well test in combination with *E. coli* expressing a genetically modified beta galactosidase MalF-β-Gal fusion. The hybrid protein MalF-β-Gal102, containing β–Gal fused to the large periplasmic domain of MalF, does not show β-Gal activity in weight cells due to the introduction of disulfide bonds, but it is active in cells that are defective in disulfide bond formation (Beckwith 2013). Among more than 50,000 screened compounds derived from two collections, six that inhibited EcDsbB and possess similar piridazinone ring structures were selected. Further in silico analysis led to identification of even more effective molecules with a similar structure. These inhibitors interfere with the interaction of EcDsbB C44 with ubiquinone. Although the researchers did not find any specific VKOR inhibitors, they documented that their strategy might be useful to search for anti-*Mycobacterium tuberculosis* drugs (Landeta et al. 2015). Adams et al. looked for molecules able to inhibit EcDsbA. They screened 1132 compounds by in vitro HTS methodology employing STD-NMR spectra and identified several potential inhibitors. The compound with greatest potency was further tested using a peptide oxidation assay and a bacterial motility test (Kurth et al. 2013). Based on the structure of this molecule, the authors inferred that it interacts with a hydrophobic groove close to the active site of EcDsbA (Adams et al. 2015). Given that homologs of DsbA share structural similarities with eukaryotic proteins of the thioredoxin family, emphasis was put on identifying inhibitors of the DsbA redox partner, DsbB. This is not easy because DsbB is an inner membrane protein with four transmembrane domains, which makes purification of fully active protein a real challenge. Fruth et al. developed a method for EcDsbB solubilization and used immobilized DsbB for a screening test. Among 1071 components in a drug-fragment library, they selected eight that interacted with EcDsbB, as validated by biochemical and biophysical methods (Fruh et al. 2010). Although this work may be a starting point to identify inhibitors of membrane proteins, work by Halili et al. showed that the data should be treated with caution, and that variation in the experimental conditions may result in different results (Halili et al. 2015). They selected one of the inhibitors identified by Furth et al. (compound number 1) and verified its activity against DsbB in a peptide oxidase assay. The rationale behind their choice concerns the compound’s function: it competes with ubiquinone for the EcDsbB binding site. Interestingly, they found that compound number 1 is a poor inhibitor of the process of electron transport. Using SAR strategy, they generated a library of several analogs of compound number 1 and examined their ability to block the oxidative Dsb pathway using various biochemical and biophysical assays. Some analogs displayed 1000 times higher activity than the starting compound. Finally, they selected one of the molecules with the greatest potency and showed that it reacts with both DsbA and DsbB (Halili et al. 2015).

Another approach took advantage of the solved structure of the covalent EcDsbA\EcDsbB complex. The work of Inaba et al. documented that a short seven-residue peptide, part of the second periplasmic loop of EcDsbB, ensures the EcDsbA/EcDsbB interaction. Further inspection of the complex structure showed that this fragment containing EcDsbB Cys104 was found inside the long cavity of EcDsbA. The EcDsbB Cys104 residue creates a disulfide bond with Cys30 of EcDsbA, and this is an indispensable element of the EcDsbA reoxidation process (Inaba et al. 2006). Based on these data, Duprez et al. synthesized several EcDsbB-derived peptides to find ones that strongly compete with native EcDsbB for EcDsbA binding. The authors confirmed binding of the slightly modified native peptide with EcDsbA and subsequently examined details of the process. They determined the structure of EcDsbA complexed with the peptide, and they used biochemical analysis of modified versions of the peptide to show the crucial role of the cysteine residue and document the influence of peptide length on efficiency of the process (Duprez et al. 2015b). Given possible anti-virulence application of this work, the same research group employed SBVS to design a more efficacious inhibitor. The solved structure of the non-covalent Pm (*Proteus mirabilis*) DsbA/heptapeptide complex was the starting point for virtual screening of a peptidomimetic library (Kurth et al. 2014). Subsequently, ten peptidomimetic compounds were examined for their binding to the hydrophobic groove of EcDsbA by biophysical and biochemical methods, but none of them displayed stronger binding than the short peptide derived from native EcDsbB (Duprez et al. 2015a).

## Conclusions

In recent years, Dsb systems of many bacterial species have been characterized. Understanding the biochemical activities of these proteins, in combination with resolution of their structures and elucidation of their influence on pathogenicity, should help combat infectious disease.
